# Supplementary material for: A Novel Non-Specific Lipid Transfer Protein Gene, CmnsLTP6.9, Enhanced Osmotic and Drought Tolerance by Regulating ROS Scavenging and Remodeling Lipid Profiles in Chinese Chestnut (Castanea mollissima Blume)
Source: Plants (Basel). 2023 Nov 20;12(22):3916. doi: 10.3390/plants12223916 (PMC10675601; doi:10.3390/plants12223916)
Supplement: Supplementary file 1 [file plants-12-03916-s001.zip › Supplementary data.pdf]

Supplementary Data

Table S1. The accession number of chestnut nsLTP family gene in NCBI.

Table S2. All the nsLTPs protein sequences of *C. mollissima, A. thaliana and B. rapa* used in this study.

Table S3. Primers used in this study.

Table S4. Lipid metabolome data in WT, CmnsLTP6.9L-OE and CmnsLTP6.9S-OE.

Table S5. Differential lipid metabolome data in WT, CmnsLTP6.9L-OE and CmnsLTP6.9S-OE.

Figure S1 Prediction of signal Peptide of CmnsLTP6.9L and CmnsLTP6.9S protein.

Figure S2 Positive detection of transgenic *Arabidopsis thaliana.* **A** CmnsLTP6.9L-OE lines by RT-PCR detection. **B** CmnsLTP6.9S-OE lines by RT-PCR detection. **C** OE lines by fluorescence detection.

Figure S3 Representation of T-DNA region of the binary vector pCAMBIA2300-EGFP used for genetic transformation.

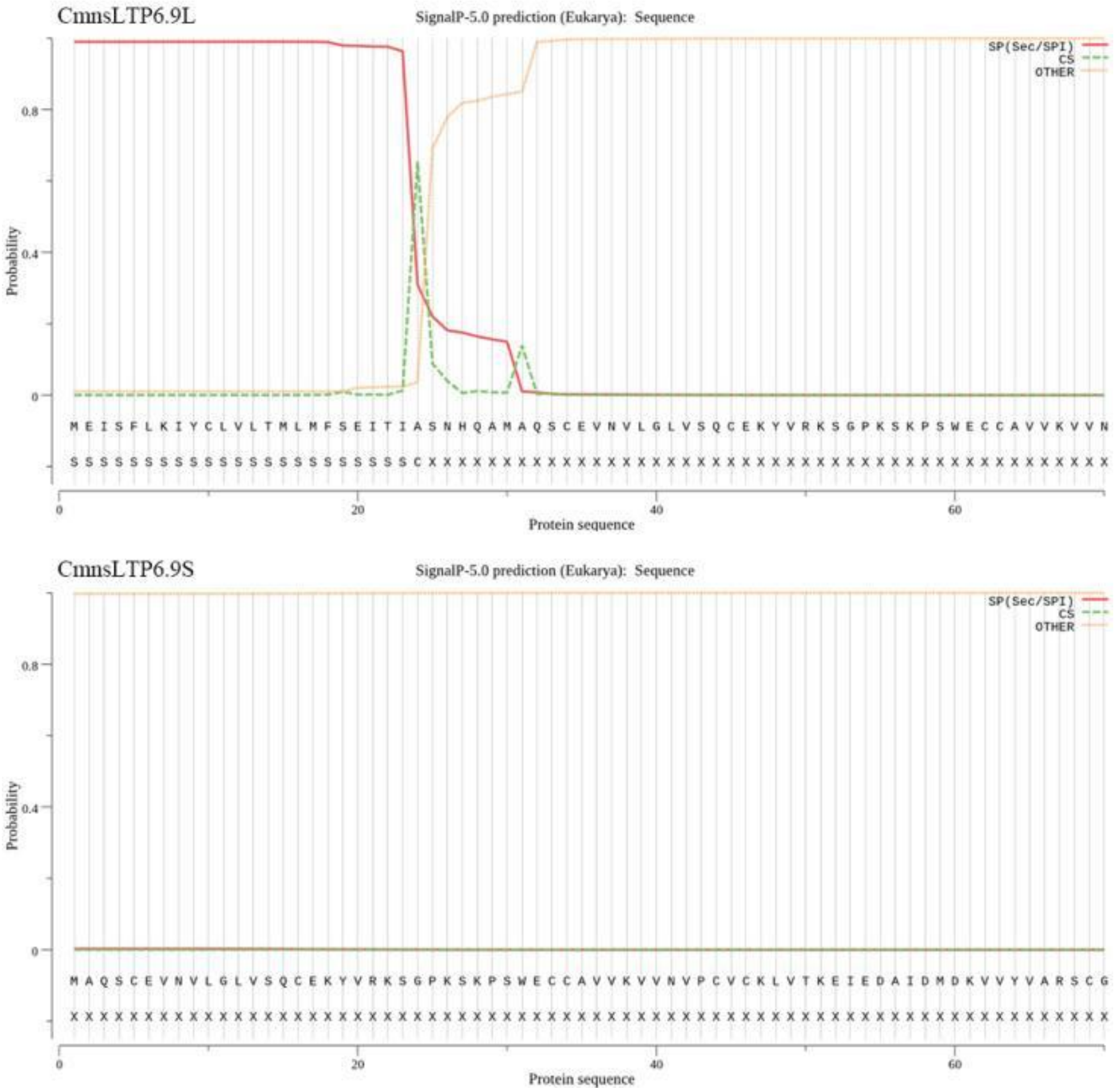

Figure S1. Prediction of signal Peptide of CmnsLTP6.9L and CmnsLTP6.9S protein.

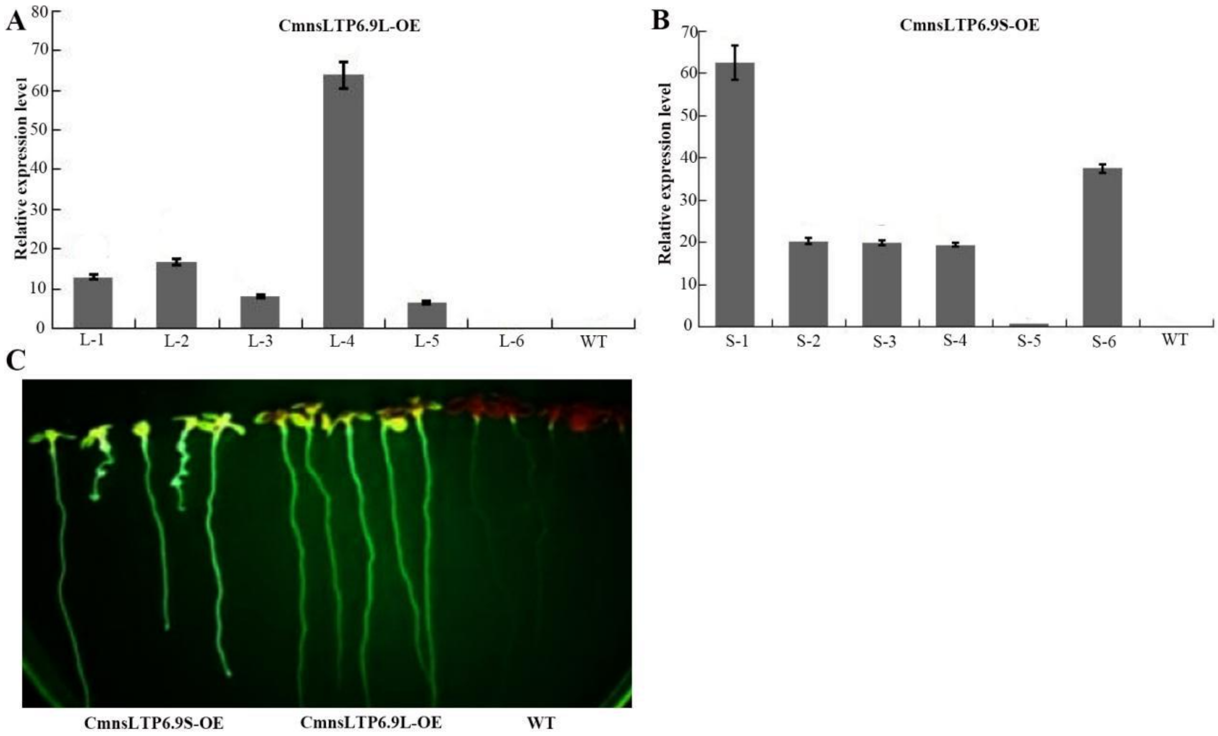

Figure S2. Positive detection of transgenic *Arabidopsis thaliana.* **A** CmnsLTP6.9L-OE lines by RT-PCR detection. **B** CmnsLTP6.9S-OE lines by RT-PCR detection. **C** OE lines by fluorescence detection.

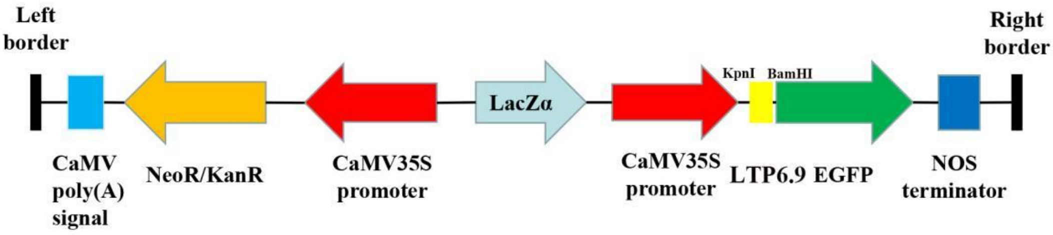

Figure S3. Representation of T-DNA region of the binary vector pCAMBIA2300-CmnsLTP6.9-EGFP used for genetic transformation.
